# Validation of undergraduate medical student script concordance test (SCT) scores on the clinical assessment of the acute abdomen

**DOI:** 10.1186/s12893-016-0173-y

**Published:** 2016-08-17

**Authors:** Matthias Goos, Fabian Schubach, Gabriel Seifert, Martin Boeker

**Affiliations:** 1 Department of General and Visceral Surgery, University Medical Center Freiburg, Hugstetter Straße 55, 79106 Freiburg, Germany; 2 Center for Medical Biometry and Medical Informatics, University of Freiburg, Stefan-Meier-Str. 26, 79104 Freiburg, Germany

**Keywords:** Clinical reasoning, Assessment, Script concordance test, Surgery, Acute abdomen, Medical education, Scales

## Abstract

**Background:**

Health professionals often manage medical problems in critical situations under time pressure and on the basis of vague information. In recent years, dual process theory has provided a framework of cognitive processes to assist students in developing clinical reasoning skills critical especially in surgery due to the high workload and the elevated stress levels. However, clinical reasoning skills can be observed only indirectly and the corresponding constructs are difficult to measure in order to assess student performance. The script concordance test has been established in this field. A number of studies suggest that the test delivers a valid assessment of clinical reasoning. However, different scoring methods have been suggested. They reflect different interpretations of the underlying construct. In this work we want to shed light on the theoretical framework of script theory and give an idea of script concordance testing. We constructed a script concordance test in the clinical context of “acute abdomen” and compared previously proposed scores with regard to their validity.

**Methods:**

A test comprising 52 items in 18 clinical scenarios was developed, revised along the guidelines and administered to 56 4^th^ and 5^th^ year medical students at the end of a blended-learning seminar. We scored the answers using five different scoring methods (distance (2×), aggregate (2×), single best answer) and compared the scoring keys, the resulting final scores and Cronbach’s α after normalization of the raw scores.

**Results:**

All scores except the single best answers calculation achieved acceptable reliability scores (>= 0.75), as measured by Cronbach’s α. Students were clearly distinguishable from the experts, whose results were set to a mean of 80 and SD of 5 by the normalization process. With the two aggregate scoring methods, the students’ means values were between 62.5 (AGGPEN) and 63.9 (AGG) equivalent to about three expert SD below the experts’ mean value (Cronbach’s α : 0.76 (AGGPEN) and 0.75 (AGG)). With the two distance scoring methods the students’ mean was between 62.8 (DMODE) and 66.8 (DMEAN) equivalent to about two expert SD below the experts’ mean value (Cronbach’s α: 0.77 (DMODE) and 0.79 (DMEAN)). In this study the single best answer (SBA) scoring key yielded the worst psychometric results (Cronbach’s α: 0.68).

**Conclusion:**

Assuming the psychometric properties of the script concordance test scores are valid, then clinical reasoning skills can be measured reliably with different scoring keys in the SCT presented here. Psychometrically, the distance methods seem to be superior, wherein inherent statistical properties of the scales might play a significant role. For methodological reasons, the aggregate methods can also be used. Despite the limitations and complexity of the underlying scoring process and the calculation of reliability, we advocate for SCT because it allows a new perspective on the measurement and teaching of cognitive skills.

## Background

Health professionals often manage medical problems in critical situations under time pressure and on the basis of vague information. Remarkably, observation of experienced clinicians making medical decisions has revealed how quickly diagnostic and therapeutic decisions are made. This complex process termed “medical problem solving” or “clinical reasoning” has been investigated for more than three decades [1].

Most surgical sub-disciplines are characterized by a high workload, high levels of stress during emergency management and in the operating theater. Therefore, clinical reasoning skills are critical in surgery. It is important to understand the underlying cognitive processes to assist students in developing clinical reasoning skills in surgical training. Furthermore, training programmes have to incorporate appropriate assessment methods [2].

In 2015, the German Society for Medical Education (GMA) and the German Council of Medical Faculties (MFT) published the National Competence Based Catalogue of Learning Objectives in Medicine (NKLM) [3]. Training the ability of undergraduate medical students to recognize and manage acute diseases of the abdomen has become an explicit goal of visceral surgical faculties.

### Clinical reasoning in surgery

In surgery, the patient presenting with acute abdominal pain requires clinical assessment and therapy within 2 h. Distinguishing between various differential diagnoses in a time-efficient way is critical and requires the “fine art of diagnostics” [4].

Clinical reasoning (CR) is considered to be one of the most important competencies of physicians [5]. This skill is known to involve analytical thought processes (type 2) as well as continuously improved intuition (type 1), the latter based on clinical experience. The dual-process theory proposes a hypothetical model to understand how these systems interact in general [6, 7]. Pattern recognition is the starting point for processing. The shortest-possible processing time is assumed as a premise. Recognized patterns lead to the diagnosis intuitively and quickly by unconscious, memory-based, and parallelized processing. Unrecognized patterns must be consciously analyzed piece-by-piece (type 2 processes), until finally type 1 processing is possible or the purely rational diagnosis is made. Type 1 and type 2 processes can oscillate and both systems have a decisive influence, possibly even negative impact – whether through irrational behavior or logical fallacies - on the diagnosis [8]. The much older script theory is largely based on the same assumptions of cognitive psychology and focusses on the type 1 operation. It still offers a far more elaborated construct for understanding how patterns (illness scripts) are formed and processed at all and offers an explanation of how experts and novices differ from each other. Thus it provides us valuable support for teaching and assessment. According to script theory, a script is an inner representation of a process, its features and the temporal order of its components [9]. In the context of medical training, script formation refers to the dynamic memorization of the typical temporal occurrence of the signs and symptoms of specific diseases. These memory units are readily retrievable and are consciously integrated in the analysis of the individual patient. Thus, they help the surgeon resolve the case at hand effectively and efficiently [6, 8, 9]. In the case of ambiguous findings, several scripts may compete with each other. In these situations, the surgeon must constantly re-assess a case and the impact of new information to prioritize scripts and proceed toward a final diagnosis.

The way in which knowledge is stored, used and retrieved characterizes the difference between novices and experts [10]. Research shows that, due to the more differentiated system of scripts readily available to them, experts use significantly less of their biomedical knowledge than novices to explain medical procedures [11]. Unfortunately, current medical specialty training too often focuses on training time, mediation of basic clinical data and knowledge without paying attention to and fostering the clinical reasoning processes involved in medical diagnosis and treatment [12, 13]. The acquisition of problem-solving and clinical reasoning abilities should begin in the early stages of medical training. Therefore, courses ought to confront students with prototypical cases [14].

Attempts to develop instruments for the global assessment of individual clinical reasoning (CR) skills have not been successful because CR is strongly context-dependent [15–17]. Thus, a student’s performance in one field need not correlate with his or her performance in another [15, 16]. Today a number of written test formats are available to reliably measure CR [18]: Key Feature tests [19], Extended-Matching Questions [20], and Short-Answer Questions [18].

The assessment methods mentioned above are based on the assumption of a rational single best response to a given clinical problem [21].

However, clinical decisions are often based on pattern recognition and goal-directed processing of illness scripts. Starting from the first encounter with a specific patient, the surgeon’s illness script delivers first diagnostic and therapeutic hypotheses, which are weighed against new information coming from history-taking, physical examination and other investigations (clinical features). Over time, this creates links between clinical features and illness, making it possible to judge the strength or weakness of a hypothesis [22]. If findings are often associated with a disease, the hypothesis is confirmed. If they are not, a hypothesis must be rejected. Script theory is now examining exactly this process.

Therefore, the SCT appears to be the most valid method, since it closely mimics clinical routine. It challenges the examinee to interpret incoming pieces of new information in a given clinical context and mark the answer on a rating scale. (See Table 1 for an example).

**Table 1:** Case of young woman, complaining of right lower quadrant pain

A 25-year old, clearly ill patient. She is brought to the ER by her husband. She complains of excruciating pain in the right lower quadrant; she has nausea, but she has not vomited.						
If you were thinking of …						
… the following diagnosis …	… and the following new information were to become …	… this hypothesis would become …				
acute appendicitis	patient vomits	−2	−1	0	+1	+2
ectopic pregnancy	the pain started suddenly two hours ago	−2	−1	0	+1	+2
ovarian torsion	Beta–HCG: 820 U/l (norm: < 5 U/l)	−2	−1	0	+1	+2
*−2: very unlikely / -1: unlikely / 0: neither likely nor unlikely / +1 more likely / +2 very likely*						
If you were considering the utility of …						
… the following treatment …	… and the following new information were to become …	… this treatment would become …				
explorative laparoscopy	mass behind the urinary bladder	−2	−1	0	+1	+2
*−2: strongly contraindicated / -1: contraindicated / 0: neither more or less indicated / +1 indicated / +2 strongly indicated*						

SCT examinees’ answers are compared to expert answer patterns, instead of comparing them to the “single best possible answer” - standard given by an examiner (e.g. MCQ). Currently the SCT is used to assess performance in specialty training as well as in undergraduate medical education [23–27].

However, there is considerable controversy in medical education literature concerning the ideal scoring procedure as well as item-based analysis for SCTs [21, 23, 27–31]. The use of the standard, so-called aggregate method, has been seriously questioned by Bland [28]. He and his colleagues suggested the distance methods as an alternative. The same authors criticize the commonly-used five-point Likert scale as being arbitrary and consider a three-point scale as sufficient. Whether these alternative scoring methods are beneficial is the subject of scientific research.

In 2010, we developed a new curriculum to foster relevant competencies and clinical reasoning, as well as an adequate assessment tool. The presented study measured clinical reasoning skills of undergraduate students who had previously followed this new curriculum in the area of the acute abdomen. The objective was to develop and validate a Script Concordance Test (SCT). Additionally, different methods of SCT scoring and item-analysis were compared.

## Methods

### The curriculum “acute abdomen” based on virtual patients

In the 4 and 5^th^ years, students at the University Medical Center Freiburg attend a 2-week mandatory visceral surgical internship. Learning performance is certified by written (MCQ) and practical examinations (OSCE).

In the routine care of patients at a university hospital, clinical training is often limited due to the lacking availability of patients with prototypical diseases during training hours. Therefore in 2010, we decided to implement virtual patients as previously described [32–35]. A new blended-learning curriculum was designed to train students in interpreting typical disease patterns, taking appropriate further diagnostic measures, making a diagnosis and ruling out competitive diagnostic hypotheses. A computer lab was built and equipped to give students access to a web-based e-learning platform (INMEDEA Simulator®, ^©^CompuGroup Medical Deutschland AG, Koblenz Germany). Students apply their knowledge in a virtual clinic, where they have the opportunity to manage surgical patients. Two 90-min seminar classes according to the think-pair-share principle [36] were held by one single surgeon with special training in teaching small groups. At the end of the internship, students took a script concordance test.

### Designing an SCT for the acute abdomen

The test was developed to assess the clinical reasoning skills of 4^th^ and 5^th^ year medical students at the end of a blended-learning seminar on the subject of acute abdomen.

We developed 18 relevant and authentic clinical scenarios and 52 test items (diagnosis and management) of varying difficulty according to a previously-described guideline [37]. The most common differential diagnoses of an acute abdomen served as a framework for the development of the cases and items in the SCT. Table 1 shows an example of an SCT case. After initial development of the cases and items, two surgeons reviewed them independently. The test material was adjusted accordingly and then submitted to an expert panel of 16 surgeons from four different teaching hospitals: 13 consultants and three experienced residents with a level of expertise between 3 and 32 years of clinical practice (mean 13.7).

Due to the novelty of content and testing method, experts were briefed on clinical reasoning and on the special features of the test.

Fifty-six students were randomly selected in the summer period of 2010 at the University Medical Center Freiburg to take the SCT after completing the internship. They, too, received prior briefing on the new test format.

### SCT scoring methods and statistical analysis

All statistical analyses were performed with Stata version 13.1 and R version 3.2.2. The statistical formulas are listed in Table 2.

**Table 2:** Formulas to calculate the raw scores

Scaletype	Method	Score
AGG	Aggregate	*p*_*i*_ = *n*_*i*_/*n*_*mode*_
AGGPEN	Wilson’s aggregate with distance penalty	*p*_*i*_ = (*pAGG*_*i*_ + *pDMODE*_*i*_)/2
DMODE	Distance to mode	*p*_*i*_ = 1 − *abs*(*i*_mode_ − *i*) * 1/*d*_*max*_ with *d*_*max*_ = 4 for 5-point Likert scales
DMEAN	Distance to mean	*p*_*i*_ = 1 − (*abs*(*ī* − *i*) * 1/*d*_*max*_ with *d*_*max*_ = 4 for 5-point Likert scales
SBA	Single best answer	$$ {p}_i=\left\{\begin{array}{cc}\hfill 1\hfill & \hfill \mathrm{f}\mathrm{o}\mathrm{r}\ {i}_{mode}\hfill \\ {}\hfill 0\hfill & \hfill \mathrm{else}\hfill \end{array}\right. $$
normalization	Z-transformation expertscale on (80, 5)	${\overline{p}}_{trans}=80+\left({\overline{p}}_{raw}-mea{n}_{exp}\right)/s{d}_{exp}*5\Big)$

**Fig. 1:**
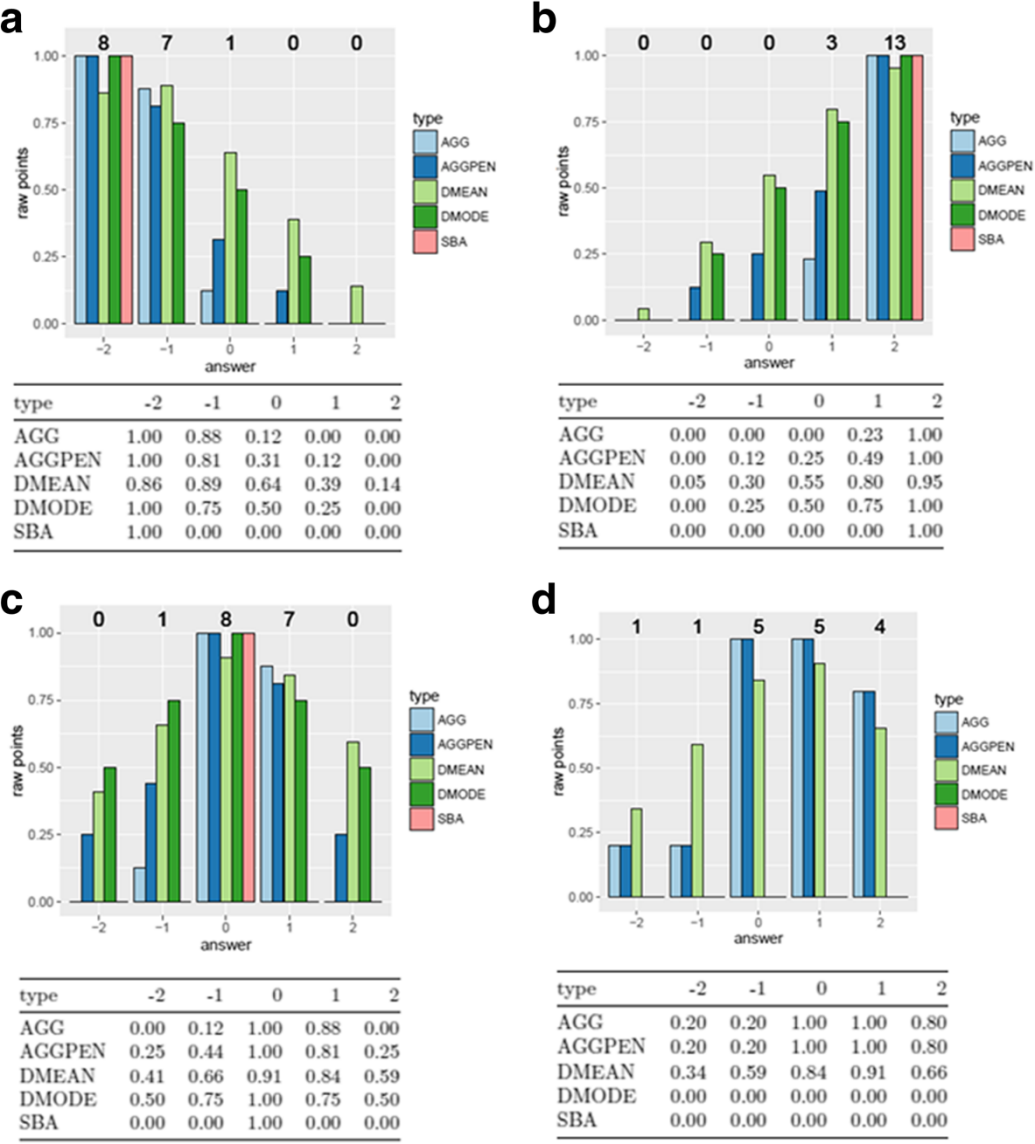
To illustrate the possible scoring of items, the calculated raw points on the basis of expert responses (bold numeral above the columns) in four selected items are shown tabular and graphic. In **a** and **b**, the expert mode is located either on the left or the right end of the scale. In **c**, the mode is in the middle of the scale, the expert answer are distributed around it. Note that, no points in the AGG scale are achieved in channels that were not selected by any expert. **d** shown an example of an item with more than one mode. In this case, the SBA could not be calculated

The aggregate scoring method (AGG) takes into account and emphasizes the distribution of expert answers. The distance method to the mean (DMEAN) and mode (DMODE) places the emphasis on the measure of central tendency and penalizes the examinee’s distance from the mean and mode expert answer respectively [28]. The aggregate with distance penalty scoring method (AGGPEN) combines the aggregate scoring method (AGG) with the distance method to the mode (DMODE) and introduces a weighted penalty for answers that differ from the mode [30]. The single best answer method (SBA) considers only the mode of the scale. All other answers except the modal score no points. Items with multiple modes are excluded from the analysis. Using an expert response panel, the various scoring methods are illustrated in Fig. 1. To keep the results comparable, a scale transformation was applied as proposed by Charlin et al. [24]. It’s based on a standard z-score and scaled so that the mean of experts is 80 with a standard distribution of five. Students’ results were easy to compare and interpret based on this scale. Generally, analysis followed the methods as reviewed in Dory et al. [38]. Item analysis was performed both on item and case basis. Items with negative item-test or item-rest correlation were excluded from the test prior to item aggregation to cases. Cases with negative item-test or item-rest correlation were deleted prior to further analysis. Scale transformation as described above was repeated after each step of item deletion to maintain the mean of the expert results at 80 with a standard deviation (SD) of five. The internal validity and reliability of an SCT is best estimated by Cronbach’s -coefficient [23].

## Results

The constructed SCT cases were derived from 18 patients (nine women and nine men), aged 7–87 years. The cases contained 52 items on 34 diagnostic hypotheses, eight treatment actions and ten investigative actions.

The item analysis was performed on item-level first followed by aggregation of the items to cases. This procedure showed slightly better results than aggregating the items to cases first with subsequent item analysis (data not shown).

**Fig. 2:**
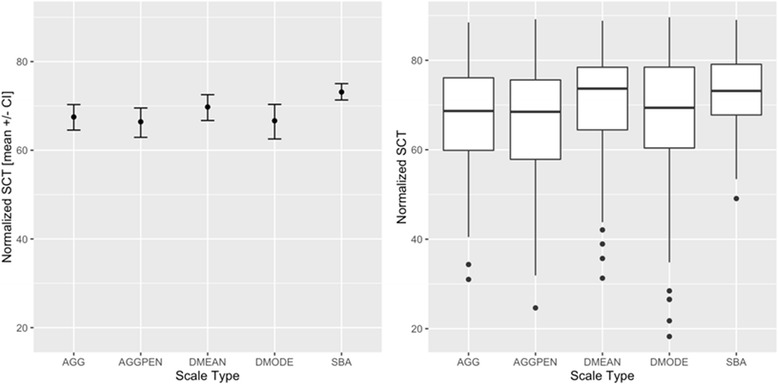
Results for different scaling methods corresponding to Table 3. Left side mean and 0.95 confidence interval of mean, right side boxplot (25, 50 and 75 percentiles)

For all scale types, the expert rating was adjusted to a mean of 80 with an SD of five as described above. The confidence interval of the mean for the expert ratings was [77.34–82.66] for all scale types. Other parameters for the expert ratings varied with the scale type. The range of the expert ratings varied from 71.9 to 88.5 with a median of 78.7 for the aggregate scale. The values for the other scales diverged slightly (Fig. 2).

Analyzed with the standard aggregate method (AGG), students scored a mean of 63.9 (CI 60.8–67.1) with a standard deviation of 11.8. This score is more than three expert standard deviations (SD = 5) lower than the expert mean of 80. The large difference clearly indicates a differentiation between the clinical reasoning skills of students and experts. The students’ results ranged from 31.0 to 83.2 corresponding to a range of ten expert SDs below to nearly one expert SD above the expert mean (Table 3).

**Table 3:** Descriptive statistics for different scaling methods

	AGG	AGGPEN	DMEAN	DMODE	SBA
Cronbach’s α	0.75	0.76	0.79	0.77	0.68
min	31.0	24.6	31.3	18.3	49.1
max	83.2	85.0	85.9	86.8	83.9
range	52.2	60.4	54.6	68.5	34.8
median	66.9	65.3	71.3	67.0	72.6
mean	63.9	62.5	66.8	62.8	71.2
SD	11.77	13.17	12.95	16.30	7.72
CI (mean, 0.95)	3.15	3.53	3.47	4.37	2.07

The median of student results for the aggregate method is 66.9. The medians for the distance to the mean (DMEAN) and the single best answer (SBA) methods are about one expert SD higher than the aforementioned. Comparing the two difference scales, the distance to the mode (DMODE) results are one expert SD smaller than distance to the mean (DMEAN). On the DMODE scale, the students’ median distance is 67.0, for the DMEAN scale it was 71.3. AGG and DMODE scale types have corresponding results. DMEAN shows best reliability measured by Cronbach’s α. Single best answer SBA has the lowest variance (Table 3).

Aggregate and distance scales are highly correlated. Best correlation was between AGG and AGGPEN with a Pearson’s correlation coefficient of 0.99 and worst correlation between DMEAN and SBA (0.81).

Internal validity (test reliability) estimated as Cronbach’s α is 0.745 for the standard aggregate method (AGG) after sequential item analysis and item deletion. Except for SBA, other scoring methods result in slightly better reliability, especially DMEAN with an α = 0.787.

There was no correlation between the SCT and the multiple choice test results. The coefficient of determination was extremely low (*R*^2^ = 0.009), which indicates that a construct independent of factual knowledge was tested for.

## Discussion

In this study, we measured the clinical reasoning skills of 4 and 5^th^ year medical students in managing the acute abdomen following their surgical internship. We constructed an SCT and designed representative cases and differential diagnoses of the acute abdomen based on current diagnostic and therapeutic guidelines [37]. Some arguments have been put forward against the content validity of SCT scoring process and estimates of panel error, which are not illuminated at this point. We refer to the work of Lineberry et al. [21]. For the purpose of this study, the SCT, which is increasingly implemented in different medical domains, is assumed to be a valid examination method that tests capabilities independent of factual knowledge. Similar to previous studies, we found no statistical correlation between standard multiple choice test results and SCT results.

Several factors have been shown to influence the reliability of SCT results [39]. The measure for the reliability of an SCT test is Cronbach’s α. However, even very good SCTs only rarely score above >0.85. One reason may be that simpler questions in a balanced SCT primarily test factual knowledge versus clinical reasoning [30, 40]. A good reliability score for SCTs typically ranges between 0.7 and 0.8 [24, 27, 40]. The goal of our study was to determine how different scoring methods and ways of optimizing data influence the reliability of surgical SCTs.

Comparative data on methods for data optimizing have not yet been presented [24, 27, 30, 31, 40]. Both question-(item-)based and case-based analyses are possible. In a summative SCT for 4 and 5^th^ year medical students e.g., question-based data optimizing resulted in an increase of reliability from 0.62 to 0.76 [27]. As expected, removal of poorly correlating items from a test improves its reliability [41]. This effect can clearly be seen in our data independent of the chosen scoring method. If the focus were only on the absolute value of Cronbach’s α, item-based data optimizing would be the method of choice. However, in the SCT, the clinical case is the appropriate measure, not the individual question. For validity,, analysis of item correlation should therefore be case-based [24], whereby this method leads to a reduction of items and thereby decreases reliability [41]. We combined the two methods in our study and sequentially performed an item-based and later case-based analysis. This sequential analysis led to a moderate increase of reliability.

The different scoring methods showed a clear effect on reliability. Various scoring methods are currently being discussed without regard for this correlation [24, 28, 30, 38]. The scoring methods differ fundamentally with respect to the numerical values attributed to experts’ answers. According to standard, SCT questions are answered on a 5-point Likert scale. The classical aggregate methods only consider answers that were also chosen by experts. Thus, examinees who correctly assess the influence but underestimate the impact of new information on a case score just as low as examinees who fail to assess both the influence and the impact of new information. This adversely affects those examinees who would be rated higher with other methods. This is also the reason why the reliability of the classical aggregate scoring methods is lower than the reliability of distance methods, which calculate a numerical score for each possible answer [28, 30]. The modified aggregate scale AGGPEN eliminates this flaw and takes into consideration the distance from the modal value. We can see this effect in an improvement of reliability (Cronbach’s α) [42]. These results correlate well with previous findings of Wilson et al., who also concluded the superiority of this method over the distance methods [30]. In our study, the reliability values didn’t reach those of the distance scales (DMEAN / DMODE). The distance scales are superior in differentiating examinees’ results. This can be seen in an increase in the range of the scores and suggests that distance methods positively affect reliability in our SCT. These results corroborate the findings of Bland et al. [28]. The distance scales used in our study barely differ from each other psychometrically. DMODE is slightly more effective in differentiating between test results than DMEAN (SD DMEAN 12.95 < SD DMODE 16.30). Taking the modal value into consideration did not improve reliability using the DMODE method.

To assign the items a single best answer based on the mode is considered as an alternative scoring method. As expected, this reduces the ability to discriminate and decreases the distance between experts and students [21, 30]. Except in SBA, reliability reached acceptable values of >0.75, allowing the generation of grades and justifying the determination of pass marks [27, 31]. Charlin’s above-mentioned scale transformation compensates the unusual, reverse scoring of the distance scales.

There is a critical point with respect to the reliability of an SCT [21]. By definition, answers to the Likert scale are not independent. Logically a single piece of new information either supports or rejects a given hypothesis, so the scale itself includes a clue. Therefore these scales are liable to reward volunteers who avoid extreme scale values and make the scores susceptible to measuring examinees response style rather than clinical knowledge [21].

In addition to the methodological difficulties in the use of the Likert scale ethnicity and culture affect the response behavior [21]. Future studies on formulation of test questions and scaling ought to clarify which scoring method is best for a given test.

Our study has some limitations due to the complex organization of the surgical curriculum in Freiburg. We were only able to include a limited number of students in the study. Although about 180 students participate in surgical training at the University Medical Center Freiburg per semester, we were only able to recruit a group of 52 students, taking into account differing educational levels and different tutors.

Future research should also address the validity of the SCTs on distinct levels of expertise, e.g. students at the beginning of their clinical training, students in the middle of their training (after the basic surgical curriculum), and those at the beginning of their postgraduate training. Furthermore, feasibility questions concerning the implementation of SCTs remain due to the necessity of expert panels and continuously updating question pools.

## Conclusion

In this study, we established an SCT in visceral surgery for the assessment of clinical reasoning skills on the topic of the acute abdomen. Results confirmed a considerable difference in clinical reasoning skills between experts and students. To our knowledge, this is the first SCT for the assessment of reasoning skills of undergraduate students in this domain. A case-based item analysis improves scale reliability less than a question-based analysis does, but should be favored over a question-based analysis due to its better validity. Concerning the scoring procedure for SCTs, our results suggest moderate superiority of the distance method over aggregate scoring. However, the considerations for content validity of each scoring method tend to favor application of the aggregation methods. Methodological limitations of the SCT-scale must be respected.

Despite the methodological limitations and complexity of the scoring and determining the reliability, we advocate for SCT because it allows a new perspective on the measurement and teaching of cognitive skills.

## Abbreviations

CR: Clinical reasoning
EMQ: Extended matching question (EMQ)
KFs: Key feature problems
MCQ: Multiple choice questions
PBA: Problem-based assessment
SAQ: Short answer question (SAQ)
SBA: Single best answer
SCT: Script concordance test
USMLE: United states medical licensing examination®
